# Swine flu-have we learnt any lesson from the past?

**DOI:** 10.11604/pamj.2015.22.118.6455

**Published:** 2015-10-12

**Authors:** Sankalp Yadav, Gautam Rawal

**Affiliations:** 1 General Duty Medical Officer-II, Chest Clinic Moti Nagar, New Delhi, India; 2 Attending Consultant, Critical Care Department, Rockland Hospital, Qutab Institutional Area, New Delhi, India

**Keywords:** Swine flu, influenza, epidemic

## Abstract

The world has suffered the pandemics due to swine flu in the past. The present epidemic in India has claimed many lives. Even, after the first outbreak of swine flu in 2009 no concrete efforts are done to prevent this infection. There is an urgent need to take radical steps to prevent such epidemics.

## Commentary

The world has faced annual epidemics and occasional pandemic, due to influenza viruses in the past [1]. The World Health Organization declared the first pandemic of the 21st century on June 11, 2009, caused by swine-origin influenza virus A (H1N1) [2, 3]. Swine origin influenza was first recognized in the border area of Mexico and United States in April 2009 [4]. The currently circulating strain of swine origin influenza virus of the H1N1 strain has undergone triple reassortment and contains genes from the avian, swine and human viruses [4–6]. Swine influenza, also called swine flu, hog flu, and pig flu, refers to influenza caused by those strains of influenza virus that usually infect pigs [2]. Swine influenza is a highly contagious acute respiratory disease of pigs [2]. Human transmission occurs by inhalation or ingestion of droplets containing viruses from people sneezing or coughing; it is not transmitted by eating cooked pork products [7]. In humans, incubation period is 2 to 7 days [4]. The symptoms of swine flu are similar to those of influenza namely chills, fever, sore throat, muscle pains, severe headache, coughing, weakness, and general discomfort [2]. Clinically, swine flu behaves similarly to seasonal flu. The only differentiating characteristics are vomiting and diarrhea in a quarter of infected patients, which are rare in seasonal flu [8]. A number of cases related to this deadly virus infection are available from all corners of the globe. The widespread influenza infection to thousands of people has resulted in substantial morbidity, medical costs, and time away from work and school [9]. India is a developing country. The present epidemic of influenza has led to fear, panic and confusion. The Government hospitals are having long queues of patients with symptoms of the swine flu. Until recently in the national capital New Delhi, only few Government hospitals were having facilities to diagnose the disease. This only resulted in the bulk inflow of the symptomatic patients to these already overburdened hospitals. India reported a total number of 5,044 reported cases of swine flu in 2012, with 405 fatalities. In 2013, there were 5,253 cases with 699 deaths. In the year 2015 till date so far 875 persons have died in various states. Also, till February 23 this year 15,413 persons have been affected by the disease [10].

However, even after repeated outbreaks in the past, there was no effective preparedness for this flu. The government has not made substantial efforts for immunizing the vulnerable population against this deadly virus. Every year teams are made to study the causes of this infection and are expected to find solutions for preventing this endemic in the future, but till date all such studies have not found any effective way to control the influenza. So far, taking precautions seems to be the only way to protect from catching this infection. But it is not easy to take precautions by the general public, as it is very difficult to educate poor and underprivileged sections of the society cover their nose and mouth while coughing and sneezing, to wash hands regularly, avoiding touching eyes, nose or mouth, avoiding close contact with sick people and staying home from work or school, if one is sick. In a country, where spitting on the roads is not a crime, it is almost impossible to control the spread of influenza just by taking precautions. The author believes that not only the poor and underprivileged who are ignorant, but the situation of the well to do families is also alike. There are also no major steps taken to inform and educate the masses about this deadly virus infection. The situation in healthcare providers is also no different. Rajoura OP et al. 2011 highlighted that there were significant gaps between knowledge and actual practice of the health care providers regarding swine flu [11]. If this is the condition of the health workers then how can we expect the general public of a low-income country where a major portion of the population are still daily wage workers or earn a very small amount, to practice healthy and hygienic attitudes? In a country where a small portion of the union budget goes to health, it is really difficult to control such epidemics. Every year, the winter begins with the same old story of rising influenza cases. The problem can't be left as it is with the hope that as the summer season will come the influenza will automatically diminish. The principal weapon against this seasonal epidemic has been effective vaccines for anyone 6 months of age or older who is at risk of becoming ill or of transmitting the viruses to others [12]. The intra-nasal vaccines are also available for children's above age of three year and adults with no known side effects, but the use of this vaccine in pregnant women and lactating mothers is not indicated [2]. However, so far no efforts have been made to immunize at-risk individuals against influenza. As per Ministry of health the virus prevalent in India is strain H1N1 and is responsive to the two antiviral agents, zanamivir (Relenza) and oseltamivir (Tamiflu), and is found to help prevent or reduce the effects of swine flu, if taken within 48 hours of the onset of symptoms. As of May 5, 2009, the Centers for Disease Control and Prevention has recommended that health care workers who provide direct care for patients with known or suspected swine influenza infection should observe contact and droplet precautions, including the use of gowns, gloves, eye protection, face masks, and fit-tested, disposable N95 respirators [13]. In addition, patients with confirmed or suspected swine influenza infection should be placed in a single-patient room with the door kept closed and airborne-infection isolation rooms with negative-pressure handling should be used whenever an aerosol-generating procedure is being performed [13]. There are many factors that contribute to the high annual incidence of influenza virus, including shortage of medicines and lack of reliable diagnostic labs. These factors should be addressed and dealt with as a priority. H1N1 infection mandates prompt clinical recognition and appropriate patient management. Proper use of rapid diagnostic tests in case finding and immediate start of treatment for the flu is essential. We cannot just wait for the winters to wane, so that the danger will diminish by itself with rising temperatures. Behavior modification is an important preventive strategy to contain the spread of H1N1 infection [11]. Besides, extensive research is essential to find ways to fight the emergence of new strains which will continue to haunt public health and the scientific communities [1].
